# Regulatory T Cells and Plasmacytoid Dendritic Cells Within the Tumor Microenvironment in Gastric Cancer Are Correlated With Gastric Microbiota Dysbiosis: A Preliminary Study

**DOI:** 10.3389/fimmu.2019.00533

**Published:** 2019-03-18

**Authors:** Zongxin Ling, Li Shao, Xia Liu, Yiwen Cheng, Chongxian Yan, Ying Mei, Feng Ji, Xiaosun Liu

**Affiliations:** ^1^State Key Laboratory for Diagnosis and Treatment of Infectious Diseases, Collaborative Innovation Center for Diagnosis and Treatment of Infectious Diseases, The First Affiliated Hospital, School of Medicine, Zhejiang University Hangzhou, China; ^2^Department of Gastrointestinal Surgery, The First Affiliated Hospital, School of Medicine, Zhejiang University Hangzhou, China; ^3^Department of Gastroenterology, The First Affiliated Hospital, School of Medicine, Zhejiang University Hangzhou, China

**Keywords:** gastric cancer, microbiota (microorganism), pDCs, Tregs (Regulatory T cells), tumor microenvironment

## Abstract

Substantial evidence indicates that gastric microbiota dysbiosis, immune system dysfunction especially immune escape are critical for gastric cancer (GC) occurrence and progression. As two important elements of tumor microenvironment (TME), the relationship between gastric microbiota and tumor-immune microenvironment is still unclear. Our present study aimed to explore the correlation between gastric mucosal microbiota in different microhabitats and its corresponding gastric immunosuppressive cells such as regulatory T cells (Tregs) and plasmacytoid dendritic cells (pDCs) in the TME. A cohort of 64 GC patients without preoperative chemotherapy was enrolled retrospectively, and 60 normal, 61 peritumoral and 59 tumoral tissues were obtained for gastric mucosal microbiota analysis and immunohistochemistry analysis. From different microhabitats, BDCA2^+^pDCs and Foxp3^+^Tregs were observed positively correlated, and increased in tumoral and peritumoral tissues compared to normal ones. The diversity, composition and function of gastric mucosal microbiota also changed more significantly in tumoral tissues than those in normal and peritumoral ones. With pearson's correlation analysis, we found that several non-abundant genera such as *Stenotrophomonas* and *Selenomonas* were positively correlated with BDCA2^+^pDCs and Foxp3^+^Tregs, respectively, while *Comamonas* and *Gaiella* were negatively correlated with BDCA2^+^pDCs and Foxp3^+^ Tregs, respectively. The increased BDCA2^+^pDCs and Foxp3^+^Tregs might be modulated by gastric mucosal microbiota, both participated in the immunosuppression microenvironment of GC, which might provide evidence to establish new strategies in antitumor therapy targeting on gastric microbiota.

## Introduction

Gastric cancer (GC) is a major health problem causing significant morbidity and mortality, ranking the third most common cause of cancer-related mortality worldwide (1, 2). Over the past decades, increasing evidence indicates that the pathogenetic mechanisms of cancer encompass tumor microenvironment (TME), emphasizing a tight correlation with its alterations for cancer initiation, progression and metastasis. The dynamic and mutualistic relationship between tumor and its TME is formed early in malignant growth and evolves throughout the life history of a tumor. TME is the primary location in which tumor cells and the host immune system interact. It is now widely accepted that the immune system can recognize and respond to tumor cells either naturally or following therapeutic intervention. The immune surveillance is considered to be an important host protection process to inhibit carcinogenesis and to maintain cellular homeostasis, while evading immune surveillance is a hallmark of biological capabilities in cancer development (3). The TME immune suppression is of the upmost importance for tumor cell survival, invasiveness and metastatic dissemination. Recently, new strategies for overcoming TEM immune suppression have led to the new and innovative cancer therapies that are considered amongst the medical breakthroughs.

As an important element of TME, human microbiota, particularly the gastrointestinal microbiota, has attracted increasing attention, as it can affect cancer growth and spread in many ways. Human microbiota has important effects on the host and that a balance of the microbiota is necessary for homeostasis. Owing to its ability to modulate host metabolism, inflammation and immunity, the human microbiota is involved in the initiation, progression and dissemination of various types of cancer both at the epithelial barriers and in sterile tissues (4–6). Growing evidence has revealed the pivotal role of the microbiota in educating and modulating the host local and distant immune system (4, 7, 8). Geva-Zatorsky et al. has shown that human gut microbiota comprises a treasure trove of immunomodulatory bacteria. With germ-free mice monocolonized with one commensal bacterial strain, they have confirmed that most these gut microbes exert several specialized, complementary, and redundant transcriptional and immunomodulatory effects by extensive, unsupervised immunophenotyping, and transcriptomics (8). The normal balanced microbiota has been proposed to have a crucial role in the establishment and maintenance of host immune homeostasis, in which the complexity of the microbial community elicits a comparably complex immunological response in the host. There is increasing evidence in animal and clinical studies that the gastrointestinal microbiota can markedly influence the clinical outcome of cancer immunotherapy, mainly mediated by shifting from immune suppression to antitumor immunity (9–11). Previous study indicated that commensal bacteria control cancer response to therapy by modulating TME, which mainly promoted an adaptive immune response against the tumors (12). So far, the gastrointestinal microbiota plays an important mechanistic role in antitumor immunity, which has implications for the use of immune checkpoint inhibitors to treat cancer.

Immune system dysfunction especially immune escape is critical for cancer occurrence and progression. Immunosuppressive cells, such as regulatory T cells (Tregs) and plasmacytoid dendritic cells (pDCs), play crucial roles in tumor immune escape (1, 13–15). The importance of forkhead box protein 3 (Foxp3) positive Tregs in mediating gastrointestinal immune homeostasis and promoting the establishment and maintenance of peripheral tolerance is evidenced in both animal and human experiments (16–18). However, in the context of cancer their role is more complex, and they are thought to contribute to the progress of many tumors (19). Although human T cells can transiently induce Foxp3 expression without acquisition of Treg function (20, 21), Ebert et al. has demonstrated that expression of Foxp3 by tumor cells is stable (22). Tregs accumulation at high density in various human carcinomas is generally associated with a poor prognosis, as expected from their capacity to inhibit antitumor immunity (23). pDCs are also one of the most important players in cancer immunity and has been identified in many solid malignant tumors (24, 25), which played an important tolerogenic role in tumor immunity. It has been shown that pDCs are recruited to solid tumor tissues and are also associated with poor clinical outcome (26, 27). Specifically, pDCs can effectively generate Tregs from naive CD4^+^ T cells, which contribute to tumor immune escape (28). Our previous study has found that Tregs and pDCs together in tumoral tissues contribute to the tumor immune escape in patients with GC (29). As mentioned above, we have learned that the gastrointestinal microbiota is essential regulator of mucosal immunity in the stomach. Specific members of the commensal microbiota, such as the genus *Clostridium* and capsular polysaccharide A (PSA)-producing *Bacteroides fragilis*, are potent inducers of Foxp3^+^Tregs (30, 31). By comparing pDCs in germ-free and specific pathogen-free mice, Swiecki et al. has found that the microbiota may promote egress of pDCs from bone marrow, which is required for homeostatic pDCs distribution (32). However, most of these studies that focused on the interplay between microbiota and the mucosal immunity were based on the intestinal environment, while these interactions in stomach were relatively understudied. How and whether the gastric microbiota in tumor and its adjacent tissues was associated with the alterations of Tregs and pDCs was still unclear. To address this issue, we analyzed the Tregs and pDCs in tumor and tumor-free tissues from the same GC patients by immunohistochemistry, and compared its gastric mucosal microbiota from normal, peritumoral and tumoral tissues by high-throughput sequencing technique. Then we explored the correlations between gastric microbiota and the two immunosuppressive cells. Our present study will provide new insights into the relationship between gastric microbiota and the tumor immunity, which will help to establish new strategies in antitumor therapy targeting on gastric microbiota.

## Materials and Methods

### Patients' Enrollment and Sample Collection

Our present study enrolled 64 GC patients without preoperative chemotherapy from April 2014 to May 2017 from our hospital. Stomach tissues were obtained from patients with primary GC who accepted gastrectomy. The tumor and tumor-free tissues were collected and confirmed by pathological diagnosis. The tumor and tumor-free (2–5 cm adjacent to the cancer tissue, Peritumor; >5 cm adjacent to the cancer tissue, Normal) tissues were collected, which were confirmed by pathological diagnosis (Table 1). The clinical and pathological staging were based on 7th edition American Joint Committee on Cancer (AJCC) cancer staging manual of GC TNM Staging (33). Finally, 59 tumoral tissues, 61 peritumoral tissues, and 60 normal tissues were selected for microbiota analysis, which were based on DNA amount and quality appropriate for 16S rRNA gene amplification and sequence analysis. The immunosuppressive cells such as Tregs and pDCs in gastric tissues were analyzed using immunohistochemistry. The following criteria were used to exclude subjects: body mass index (BMI = weight in kilograms divided by the height in meters squared) >30; use of antibiotics, probiotics, prebiotics, or synbiotics in the previous month; preoperative chemotherapy, radiotherapy, or other biological treatment before gastrectomy. The fresh stomach tissues were obtained from surgery and stored at liquid nitrogen immediately until further use.

**Table 1:** Clinical parameters of the patients^*^.

**Characteristics**	**Patients (*n* = 64)**
Age (year, means ± SD)	60.30 ± 12.75
Gender (Female/Male)	24/40
BMI (means ± SD)	22.37 ± 3.25
Tumor localization, no	
Proximal stomach	5
Body/Fundus	34
Antrum	25
Tumor differentiation, no	
High differentiated	0
Moderately/poor differentiated	64
Tumor stage, no	
I (Ia, Ib)	5
II (IIa, IIb)	8
III (IIIa, IIIb, IIIc)	47
IV	4
HP infection, no	
Positive	64
Negative	0
Antibiotics use, no	0
PPI use, no	64
Pre-operative chemotherapy, no	0
Sample collection	
Normal, no	60
Peritumor, no	61
Tumor, no	59

* *BMI, Body mass index; HP, Helicobacter pylori; no, number; PPI, Proton pump inhibitors; SD, standard deviation*.

### Immunohistochemistry (IHC)

Blood dendritic cell antigen-2 (BDCA2) and Foxp3 were selected as cell markers of pDCs and Tregs, respectively. The expression of these immunosuppressive proteins in tissues was evaluated via immunohistochemical analysis. Serial 5-μm frozen sections were used in this study. The details of the IHC procedure were performed as described in our previous studies (29, 34). Commercially available primary antibodies were used according to the manufacturer's instructions (mouse anti-BDCA2, 1:800 dilution, MACS, CA; mouse anti-Foxp3, 1:200 dilution, Abcam, Cambridge, MA, USA). Negative control staining was carried out with cold PBS in place of primary antibody. Numbers of BDCA2^+^pDCs and Foxp3^+^Tregs in each of 5 high-power fields (magnification 400 ×) were counted by two blinded, independent researchers using a microscope (objective lens, 40 ×; ocular lens, 10 ×; BX41 microscope, Olympus, Japan).

### Gastric Mucosal Bacterial DNA Isolation, Amplicon Library Construction and Sequencing

Gastric mucosal bacterial genomic DNA was extracted from tissue samples by using the QIAamp DNA Mini Kit (QIAGEN, Hilden, Germany) according to the manufacturer's instructions with minor modifications (35). Amplicon libraries were constructed with Illumina sequencing-compatible and barcode-indexed bacterial PCR primers 319F/806R, which target the V3-V4 regions of 16S rRNA gene (36). All PCR reactions were performed with KAPA HiFi HotStart ReadyMix using the manufacturer's protocol (KAPA Biosystems) and approximately 50 ng of extracted DNA per reaction. Thermocycling conditions were set at 95°C for 1 min, 55°C for 1 min, then 72°C for 1 min for 30 cycles, followed by a final extension at 72°C for 5 min. All PCR reactions were performed in 50 μl triplicates and combined after PCR. The amplicon library was prepared using a TruSeq^TM^ DNA sample preparation kit (Illumina Inc, San Diego, CA, USA). Prior to sequencing, the DNA concentration of each PCR product was extracted with the MiniElute® Gel Extraction Kit (QIAGEN) and quantified on a NanoDrop ND-1000 spectrophotometer (Thermo Electron Corporation) and Qubit 2.0 Fluorometer (Invitrogen). The purified amplicons were then pooled in equimolar concentrations and the final concentration of the library was determined by Qubit (Invitrogen). Negative DNA extraction controls (lysis buffer and kit reagents only) were amplified and sequenced as contamination controls. Sequencing was performed on a MiSeq instrument (Illumina) using a 300 × 2 V3 kit together with PhiX Control V3 (Illumina).

### Bioinformatic Analysis

The 16S rRNA gene sequence data set generated from the MiSeq run were first merged and demultiplexed into per samples using the QIIME version 1.9.0 with default parameters (37). Chimera sequences were detected and removed using the USEARCH software based on the UCHIME algorithm (38). Open-reference operational taxonomic unit (OTU) pick was then performed with USEARCH V7 referenced against Greengenes database version 13.8 at 97% sequence similarity (39, 40). OTUs with a number of sequences <0.005% of the total number of sequences were discarded as recommended (41). The result was an OTU table, which could be used for subsequent downstream analysis.

For taxonomic assignment, the most abundant sequences were chosen as the representative sequences of corresponding OTUs. Taxonomic assignment of individual datasets were classified against the Greengenes database version 13.8 using both RDP classifier and UCLUST version 1.2.22 methods implemented in QIIME (39, 42). Any sequences that were identified as members of Eukarya, Archaea, Mitochondria, Chloroplasts and Cyanobacteria lineages, were removed. Alpha diversity was calculated with QIIME software with Python scripts base on the sequence similarity at 97% level, including index of observed species (OTU number), abundance-based coverage estimator (ACE), Chao1 estimator, Shannon, Simpson, Evenness and PD whole tree. Sequence coverage was assessed in mothur by rarefaction curves and Good's coverage (43, 44). Beta diversity was measured by unweighted and weighted UniFrac distance calculated with 10 times of subsampling by QIIME. These distances were visualized by principal coordinate analysis (PCoA) (45). Hierarchical clustering was performed and heatmap was generated using a Spearman's rank correlation coefficient as a distance measure and a customized script developed in the R statistical package. The output file was further analyzed using Statistical Analysis of Metagenomic Profiles software package (STAMP) version 2.1.3 (46).

For the predictive functional analyses, PiCRUSt software package version 1.0.0 was used to identify predicted gene families and associated pathways from inferred metagenomes of taxa of interest identified from the compositional analyses, which was based on the fact that phylogeny and function are closely linked (47). Predicted functional genes were categorized into Kyoto Encyclopedia of Genes and Genome (KEGG) orthology (KO), and compared across patient groups using STAMP at KEGG level 3. Pathways and enzymes were assigned using KEGG database options built into the pipeline. The pathways that were non-prokaryotic, had fewer than 2 sequences in each cohort, or had a difference in mean proportions <0.1% were excluded from analysis. The characterization of microorganismal features differentiating the gastric microbiota was performed using the linear discriminant analysis (LDA) effect size (LEfSe) method (http://huttenhower.sph.harvard.edu/lefse/) for biomarker discovery, which emphasizes both statistical significance and biological relevance (48). With a normalized relative abundance matrix, LEfSe uses the Kruskal-Wallis rank sum test to detect features with significantly different abundances between assigned taxa and performs LDA to estimate the effect size of each feature. A significant alpha at 0.05 and an effect size threshold of 2 were used for all biomarkers discussed in this study.

### Statistical Analysis

For continuous variables, independent *t*-test, White's non-parametric *t*-test, and Mann-Whitney *U*-test were applied. For categorical variables between groups, Pearson chi-square or Fisher's exact test was used, depending on assumption validity. For taxon among subgroups, ANOVA test was applied (Tukey-Kramer was used in *post-hoc* test, Effect size was Eta-squared). For correlation analyses, Spearman's rank correlation test was used. False-discovery rate (FDR) was calculated according to Benjamini-Hochberg, FDR-corrected *p*-values were denoted as Q_FDR_ and was used when performing all untargeted screening analyses of different taxa. Statistical analysis was performed using the SPSS V19.0 (SPSS Inc., Chicago, IL) and STAMP V2.1.3 (46). Correlations between immunosuppressive cells and key functional bacteria were assessed by calculating the Pearson correlation coefficient (*r*). GraphPad Prism version 6.0 (San Diego, CA) was used for preparation of graphs. All tests of significance were two sided, and *p* < 0.05 or corrected *p* < 0.05 was considered statistically significant.

### Accession Number

The sequence data from this study were deposited in the GenBank Sequence Read Archive with the accession number SRP172818.

## Results

### Increased BDCA2^+^pDCs and Foxp3^+^Tregs in Tumoral and Peritumoral Tissues

**Figure 1:**
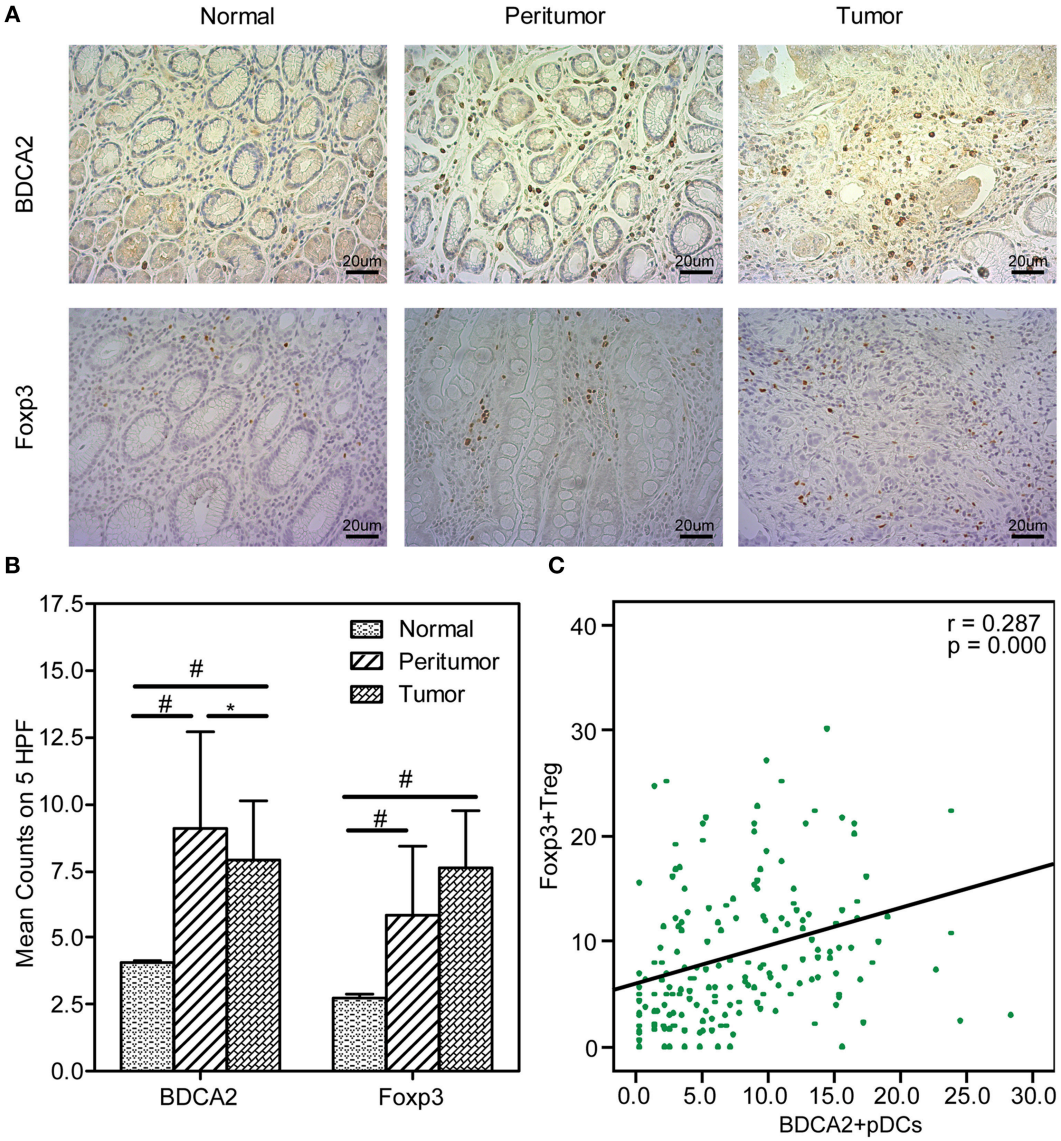
Increased BDCA2^+^pDCs and Foxp3^+^Tregs in tumoral and peritumoral tissues. Representative immunohistochemical (IHC) staining of BDCA2 and Foxp3 in tumoral, peritumoral, and normal gastric tissue are shown (magnification, × 400) **(A)**. Box plot set showing the BDCA2^+^pDCs and Foxp3^+^Treg cell level in different tissues. Independent *t-*tests were used to analyze variation among the three stomach microhabitats. #*p* < 0.01 when compared normal group with peritumoral and tumoral groups; ^*^p < 0.05 when compared peritumoral group with tumoral groups **(B)**. The presence of BDCA2^+^pDCs was positively correlated with the increased number of Foxp3^+^Tregs in stomach tissues **(C)**.

BDCA2^+^pDCs and Foxp3^+^Tregs were two major immunosuppressive cells in TME. BDCA2^+^pDCs are a relatively rare DC subset in tissues, while Foxp3 is the best-known marker for evaluating real Tregs that have a suppressive function. Immunohistochemical staining of BDCA2 revealed that the absolute numbers of pDCs were present in stomach peritumoral and tumoral tissues of GC patients, and Foxp3 indicated that Tregs were also increased than those in normal tissues (Figure 1A). We observed that increased number of BDCA2^+^pDCs in peritumoral (11.68 [range, 1.60–27.60], *p* < 0.001) and tumoral tissues (9.51 [range, 0–30.20], *p* < 0.001), when compared with the normal tissues (4.14 [range, 0–24.75]). We also found that the numbers of BDCA2^+^pDCs in tumoral tissues were significantly lower than that in peritumoral tissues (*p* < 0.05). In addition, the number of Foxp3^+^ Tregs in tumoral tissues (9.12 [range, 1.00–28.60]) were higher than that in peritumoral tissues (7.68 [range, 1.00–15.40], *p* < 0.001), and three times that in normal gastric mucosa (2.83 [range, 0.00–14.80], *p* < 0.001) (Figure 1B). We found that the prevalence of pDCs was significantly associated with that of Tregs in gastric tissues (Figure 1C; *r* = 0.287, *p* = 0.000). These results indicated that increased numbers of pDCs and Tregs in stomach tissues of GC patients might contribute to the TME immune suppression of GC.

### Altered the Diversity and Composition of Gastric Microbiota in TME

**Figure 2:**
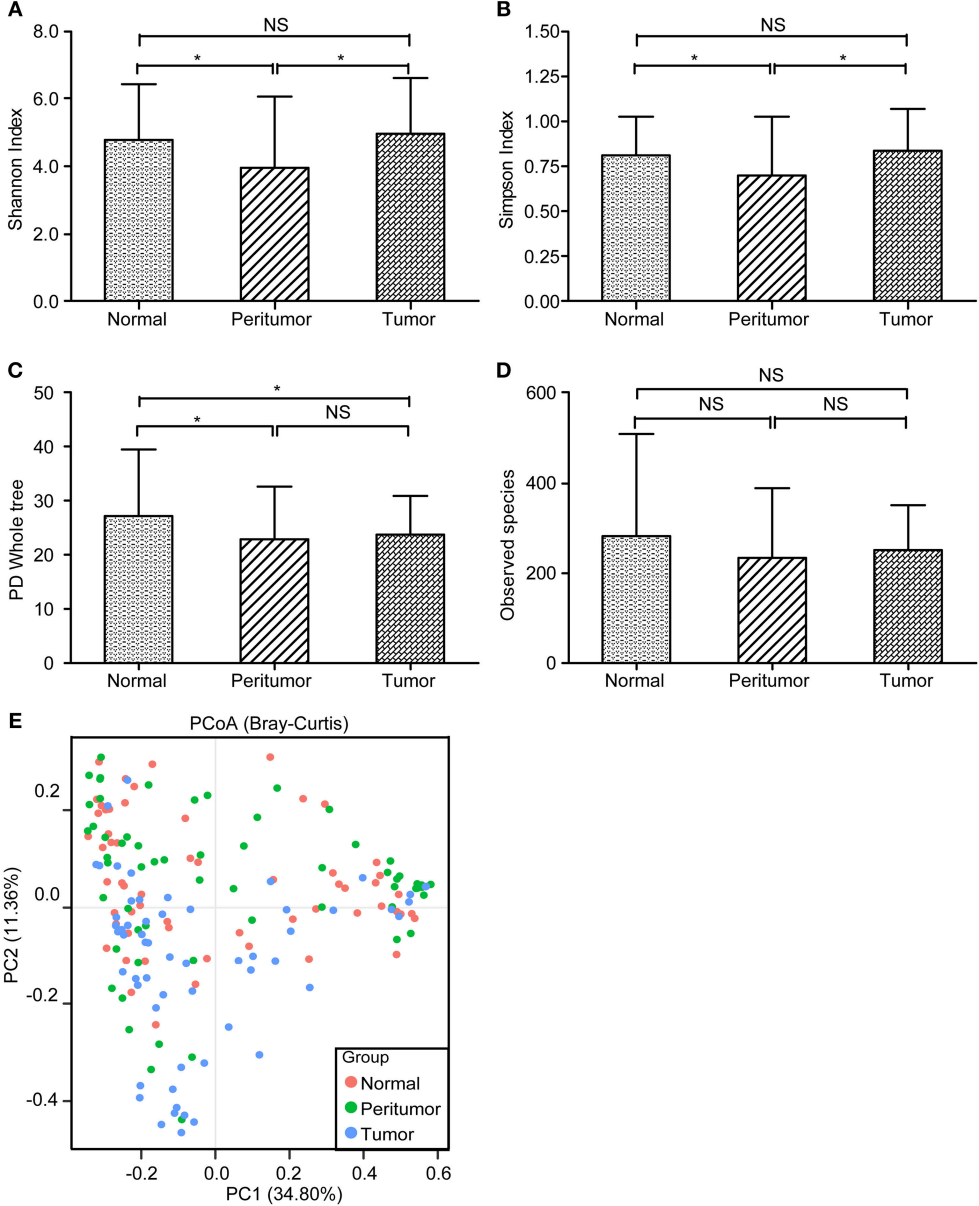
The diversity and richness of the gastric microbiota in different stomach microhabitats. Diversity indices such as Shannon **(A)** and Simpson **(B)**, and richness indices, such as PD whole tree **(C)** and observed species (OTU number, **D**), were used to evaluate the overall structure of the gastric microbiota in the three stomach microhabitats. The data are presented as the mean ± standard deviation. Unpaired *t*-tests (two-tailed) were used to analyze variation among the three stomach microhabitats. Principal coordinate analysis (PCoA) plots of individual gastric mucosal microbiota based on Bray-Curtis distance **(E)**. Each symbol represents a sample. ^*^p < 0.05; NS, no significant.

With MiSeq platform, totally 10,099,372 high-quality reads with an average of 56,107 reads per sample were used for following analysis, with 3,283,689 reads in normal group, 3,591,578 reads in peritumoral group and 3,224,105 reads in tumoral group, respectively. The Good's coverage was 99.98%, indicating that almost all bacteria in the stomach have been obtained. The diversity indices such as Shannon and Simpson were significantly increased in peritumoral group, when compared with normal and tumoral groups (Figures 2A,B; *p* < 0.05), while the richness indices such as PD Whole tree but not observed species was significantly decreased in peritumoral and tumoral groups when compared with normal group (Figures 2C,D; *p* < 0.05). However, PCoA based on Bray-Curtis distance could not separate the three groups into different clusters (Figure 2E).

**Figure 3:**
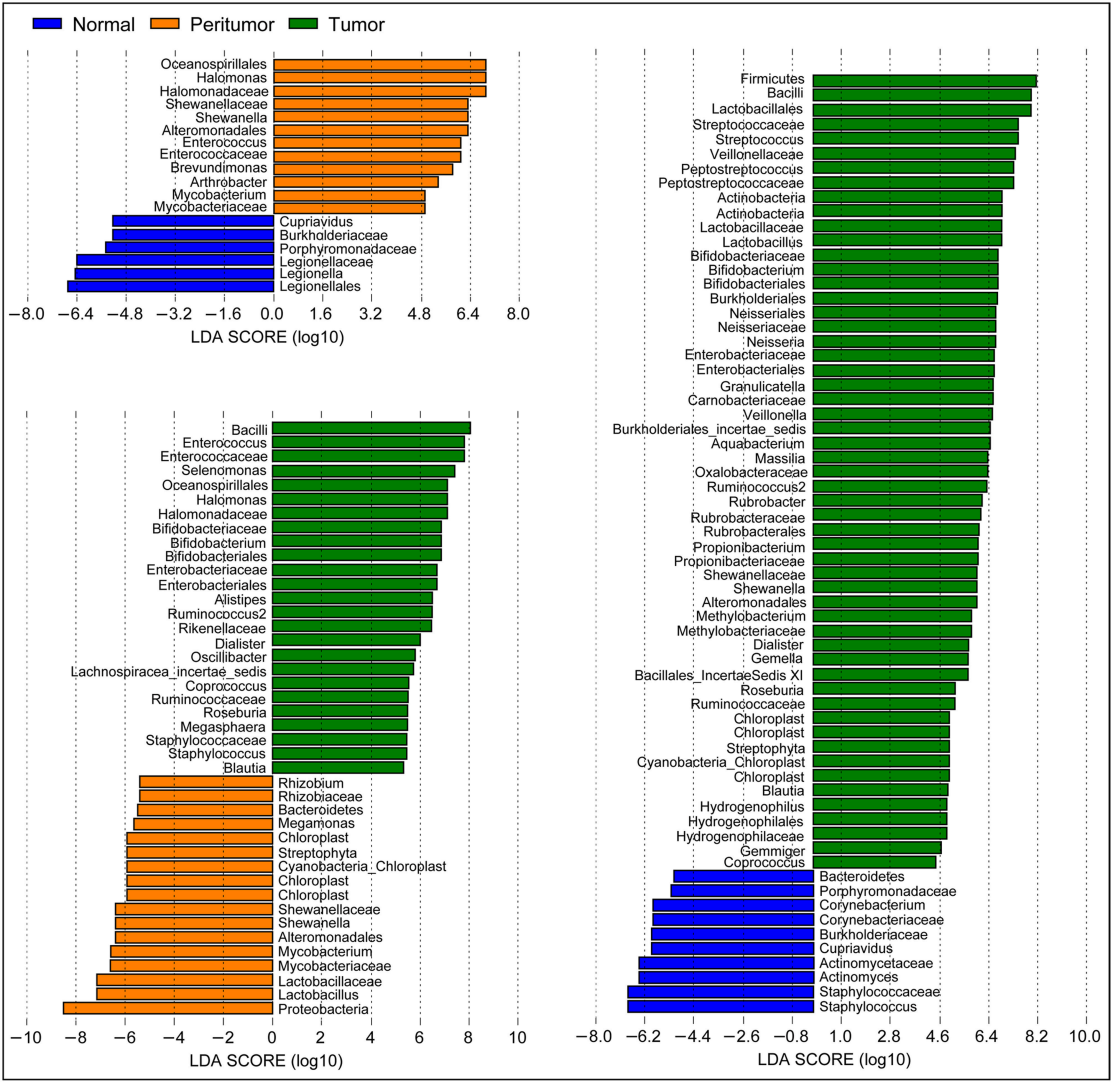
Different bacterial taxa among the three stomach microhabitats. LEfSe identifies the taxa with the greatest differences in abundance among the three stomach microhabitats. Only the taxa meeting a significant LDA threshold value of >2 are shown.

LEfSe has found that Proteobacteria, Firmicutes, Bacteroidetes, Actinobacteria, Acidobacteria, and Fusobacteria were the dominant phyla in stomach. Our present data indicated that Proteobacteria was enriched in peritumoral group, while Firmicutes and Fusobacteria were enriched in tumoral group (*p* < 0.05). However, the abundant phyla, Bacteroidetes, and Actinobacteria, were not changed significantly among the three groups. In present study, we found that the composition of the gastric microbiota in tumoral group changed dramatically. At the genus level, we found that the only several abundant genera such as *Halomonas, Shewanella, Enterococcus*, and *Brevundimonas* were increased significantly and *Legionella* was obviously decreased in peritumoral group when compared with normal group (Figure 3; LDA score>2; *p* < 0.05). However, more abundant genera such as *Streptococcus, Peptostreptococcus, Lactobacillus, Bifidobacterium, Neisseria, Veillonella*, and *Shewanella* were increased, while *Staphylococcus* and *Corynebacterium* were decreased significantly in tumoral group when compared with the normal group. We also found that *Enterococcus, Selenomonas, Halomonas, Bifidobacterium, Dialister, Roseburia*, and *Staphylococcus* were increased significantly and *Lactobacillus, Shewanella*, and *Megamonas* were decreased in tumoral group when compared with peritumoral group. Interestingly, the GC associated pathogen, *Helicobacter pylori* (HP), was increased significantly in peritumoral group but not in normal and tumoral groups (*p* < 0.05). However, microbial biomarkers discovery using LEfSe found that the LDA score value was not higher than 2 fold for HP in our present study.

### Correlations Between Gastric Microbiota and Immunosuppressive Cells in TME

**Figure 4:**
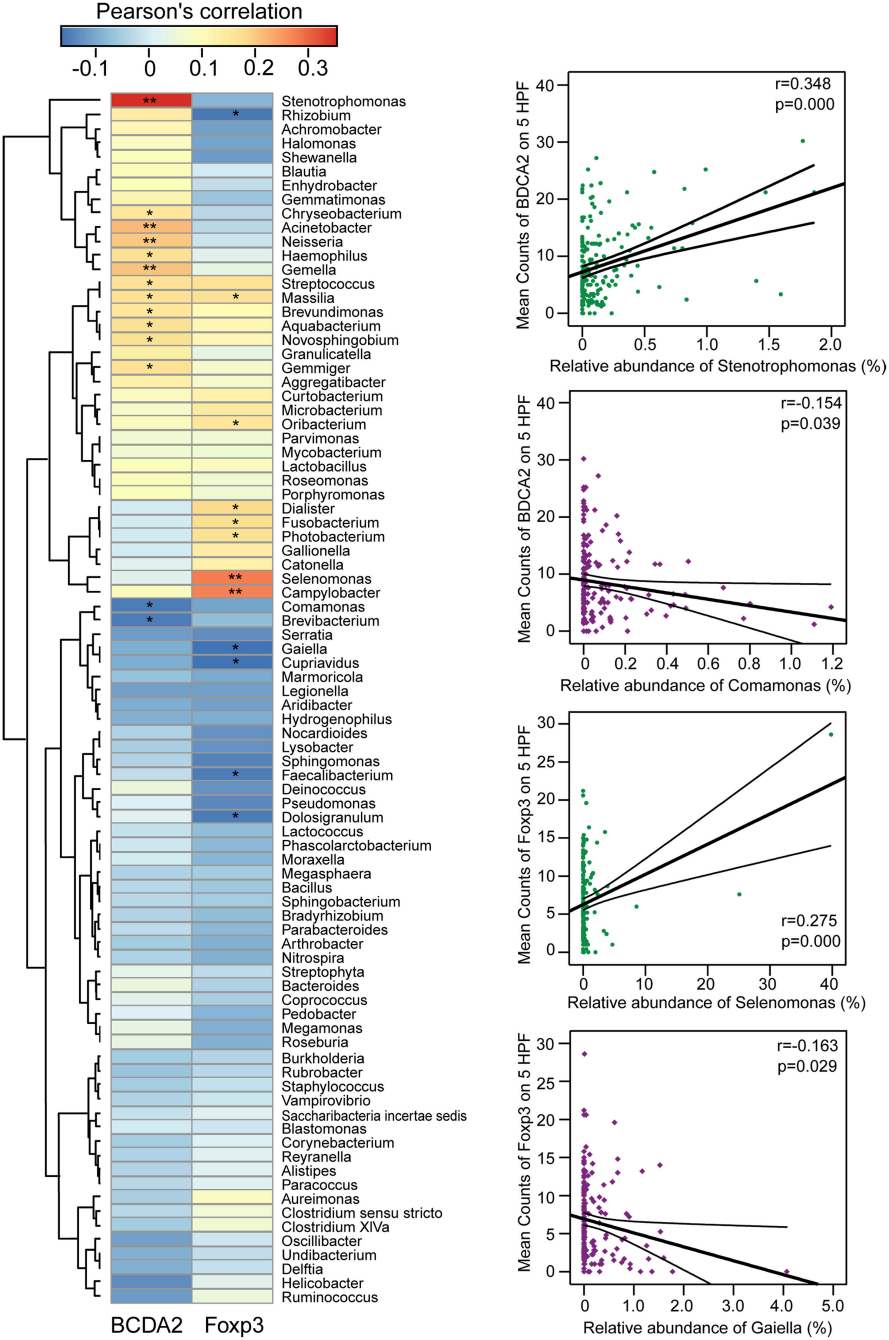
Correlations between gastric microbiota and immunosuppressive cells in the TME. The pearson's correlation between gastric mucosal microbiota and two major immunosuppressive cells such as BDCA2^+^pDCs and Foxp3^+^Tregs in the TME was shown in the heatmap. The four genera such as *Stenotrophomonas, Comamonas, Selenomonas*, and *Gaiella* were shown their correlation with BDCA2^+^pDCs and Foxp3^+^Tregs in details. ^*^p < 0.05; ^**^p < 0.01.

We explored potential correlations between the gastric mucosal bacterial populations and two major immunosuppressive cells in TME. With Pearson's correlation analysis, we found that *Stenotrophomonas, Acinetobacter, Gemella, Neisseria, Aquabacterium, Haemophilus, Novosphingobium, Streptococcus, Massilia, Gemmiger, Chryseobacterium*, and *Brevundimonas* were positively correlated with BDCA2^+^pDCs, while *Comamonas* and *Brevibacterium* were negatively correlated with BDCA2^+^pDCs in the Heatmap (*p* < 0.05; Figure 4). In addition, the genera of *Streptococcus, Massilia, Oribacterium, Campylobacter, Selenomonas, Dialister, Photobacterium*, and *Fusobacterium* were positively correlated with Foxp3^+^ Tregs, while *Rhizobium, Gaiella, Cupriavidus, Faecalibacterium*, and *Dolosigranulum* were negatively correlated with Foxp3^+^ Tregs (*p* < 0.05). Notably, most of these bacteria that significantly correlated with immunosuppressive cells in TME were non-abundant bacteria. In details, *Stenotrophomonas* was positively correlated with BDCA2^+^pDCs (*r* = 0.348; *p* = 0.000) and *Comamonas* was negatively correlated with BDCA2^+^pDCs (*r* = −0.154; *p* = 0.039), while *Selenomonas* was positively correlated with Foxp3^+^Tregs (*r* = 0.275; *p* = 0.000) and *Gaiella* was negatively correlated with Foxp3^+^Tregs (*r* = −0.163; *p* = 0.029). However, no significant correlations were observed between *Helicobacter* and the two major immunosuppressive cells in TME (*p* > 0.05). These correlations between the gastric mucosal bacteria and the two major immunosuppressive cells might contribute to the immune system dysfunction in TME.

### Inferred Functional Changes of GC-Associated Gastric Mucosal Microbiota in TME

**Figure 5:**
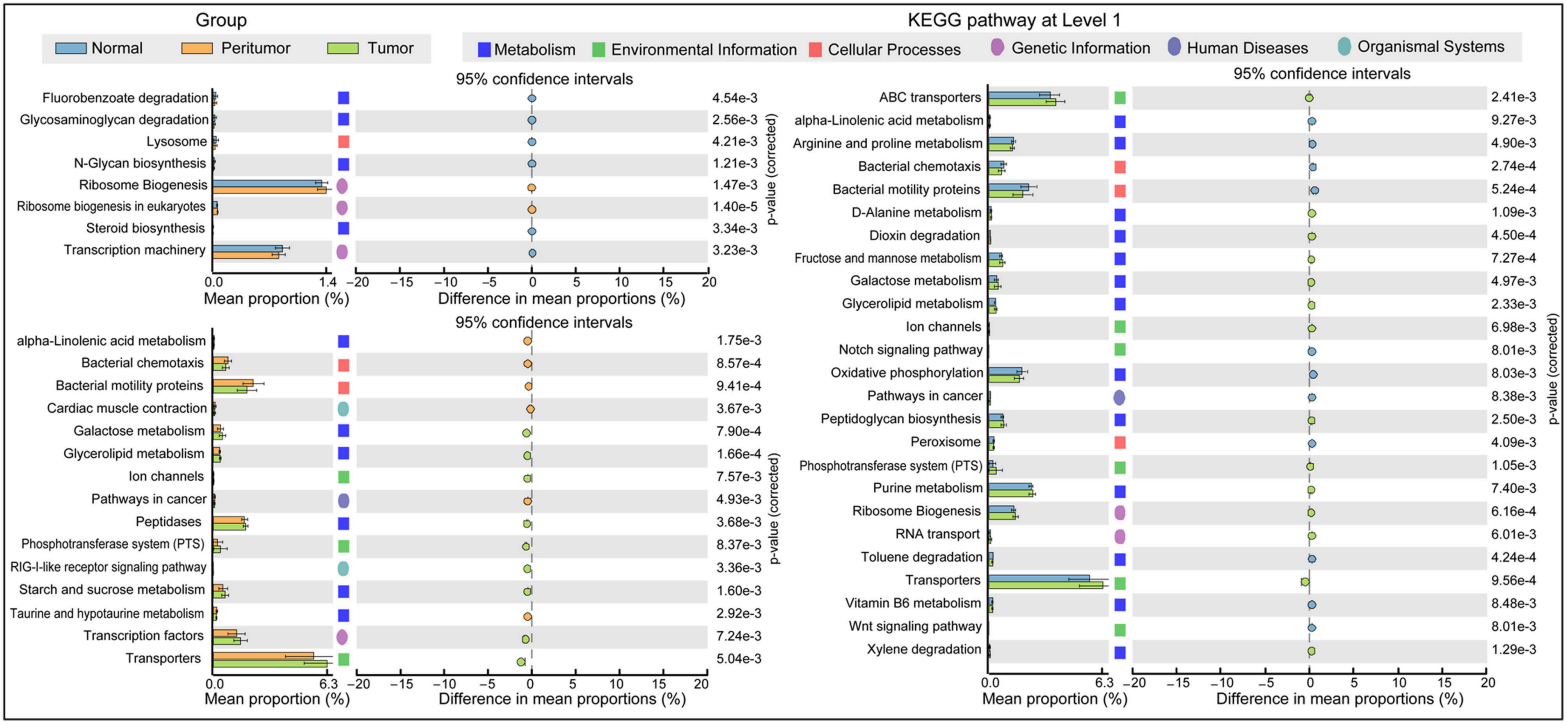
PiCRUSt-based gastric microbiome study in the three different stomach microhabitats. The different bacterial functions in the three stomach microhabitats were evaluated between each other based on two-sided Welch's *t*-test. Comparisons among the three stomach microhabitats for each KEGG pathway at level 3 were shown by percentage. The Benjamini–Hochberg method was used for multiple testing correction based on the false discovery rate (FDR) by STAMP.

The functional content of the gastric microbiota was predicted by PiCRUSt based on closed-reference OTU picking. We utilized STAMP (statistical analysis of taxonomic and functional profiles) to analyze differences at the KEGG level 3, a database for understanding metabolic functions, and at the gene level (Welch's *t*-test, Benjamini-Hochberg adjustment). In our present study, there were greater gastric microbiota associated KEGG gene function changes in tumoral group than those in normal and peritumoral groups, which was similar with the changes of gastric microbiota mentioned above. At KEGG level 3 that included 328 KEGG pathways, we identified eight different KEGG pathways that were significantly different between normal and peritumoral groups, 15 different KEGG pathways that were significantly different between peritumoral and tumoral groups, and 25 different KEGG pathways that were significantly different between normal and tumoral groups (Q_FDR_ < 0.05, Figure 5). These differential KEGG pathways mainly belonged to metabolism-related pathways, environmental information processing pathways, cellular processing pathways, genetic information processing pathways, human diseases-related pathways and organismal systems-related pathways. Nine and nineteen differential KEGG pathways predicted to be significantly upregulated in tumoral group, while 6 and 11 differential KEGG pathways were significantly downregulated when compared with peritumoral and normal groups, respectively. Several metabolism-related pathways were upregulated simultaneously in both peritumoral and tumoral groups, which might contribute to the GC TME changes.

## Discussion

Growing evidence indicates that complex interactions among cells and their microenvironment is critical for both normal tissue homeostasis and tumor growth. TME has many components such as cells, soluble factors, signaling molecules, extracellular matrix and mechanical cues that can promote neoplastic transformation, support tumor growth and invasion, protect the tumor from host immunity, foster therapeutic resistance, and provide niches for dormant metastases to thrive (49). Although the importance of microenvironmental alterations in tumor development is recognized, the molecular mechanisms underlying these changes are only now beginning to be understood. Recently, human microbiota that thrive on and within the human habitats has been considered as one of the important elements of TME, which have been implicated in the development of some cancers including GC. Mounting evidence indicates that the gut microbiota affects inflammation and immunity not only locally at the mucosal level but also systemically, which contributes to initiation, progression, metastasis and even treatment of cancer. In human stomach, the gastric mucosal microbiota has recently been investigated by high-throughput sequencing techniques, which showed that the gastric microbiota is more complex than previously appreciated. Considerable progress has been made toward understanding the altered composition and diversity of the gastric microbiota in gastric diseases (50–58), which has provided evidences that bacteria, including members of Proteobacteria, Firmicutes, Actinobacteria, and Fusobacteria phyla, can be regularly detected in gastric biopsies with gastric microbial dysbiosis associated with GC. Of course, the inter-personal variations among the stomach microbiota could not be ignored. The GC are multifactorial pathologies involving changes in the gastric microbiota, which can affect inflammatory processes of the stomach TME that contribute to cancer and its therapy. In human, defining inflammation and the presence of inflammatory cells within or surrounding the tumor is a critical aspect of modern pathology. Given the lack of understanding of the impact of inflammation on the progression of GC, it would be premature to design clinical therapeutic strategies that target inflammation as an approach to treating established cancers at the present time. However, numerous experimental studies suggest that changed immune response such as inflammatory cells and their associated mediators that regulated by gut microbiota form a microenvironment favoring the development of colorectal cancer, presumably by enhancing DNA damage in epithelial cells (59–61). Recent advance has indicated that cancer immunotherapy targeting these altered immune profiles has emerged as a powerful and promising clinical approach for treatment of cancer. The intimate interactions between host microbiota and immunological responses in TME may shed light on novel anticancer therapeutic strategies by manipulating human microbiota.

Traditionally, many previous studies have always considered the whole stomach as one habitat (50–58). However, the stomach microhabitats are not uniform; rather, they vary considerably across sites within the stomach, at the same site over time, and with health status. Different stomach microhabitats might determine different gastric microbiota and different gastric mucosal immunity, which formed quite distinct TME. In our present study, we investigated the connection between gastric microbiota and immunological responses especially immune escape in specific gastric microhabitats for the first time. Our present study found that higher relative abundance of the gram-negative phylum of Fusobacteria was associated with TME. Previous study has shown that *Fusobacterium nucleatum* (belonged to Fusobacteria phylum) can express the Fap2 protein that is able to interact directly with TIGIT (T-cell immunoglobulin and ITIM domain), an immune checkpoint marker that can be expressed on T cells in the TME, thus influencing patients' prognoses (62). In fact, it was more difficult to obtain healthy stomach tissues as controls; therefore, we screened confirmed GC tumoral, and peritumoral tissue, and neighboring normal tissue from the same GC patient to investigate discrepancies of gastric microbiota and its mucosal immunity in the TME, which could effectively screen key players for GC development.

From the three stomach microhabitats, the two major immunosuppressive cells, BDCA2^+^pDCs and Foxp3^+^Tregs, are significantly higher in peritumoral and tumoral tissues than those in normal ones. DCs are the most potent professional antigen-presenting cells of the immune system, either boost the immune system (enhancing immunity) or dampen it (leading to tolerance), depending on their maturation status (63). This dual effect explains the dual outcomes of cancer progression. Numerous experimental and clinical evidence shows that one DC subtype, pDCs, possess immunosuppressive and tolerogenic property and, thus, promote tumor growth and progression, which has been found to infiltrate in various cancer including GC (29, 64). Consistent with our previous study, we also found that pDCs preferentially accumulated in peritumoral tissue rather than that in tumoral ones (*p* < 0.05). This distribution bias has also been reported in cervical neoplastic lesions, in that pDCs were mainly detected in the stroma underlying the tumor rather than within the cancerous epithelium (65). BDCA2 is a novel type II C-type lectin presumably involved in ligand internalization, processing and presentation, as well as in inhibition of α/β-interferon (IFN-α/β) synthesis in pDCs (66). Tel et al. has demonstrated that immature pDCs can be readily detected by their expression of BDCA2, acting as a hallmark of human pDCs, which is down-regulated when pDCs mature (67), despite the latest finding conducted by Vescovi et al. has shown that BDCA2 staining remains detectable in toll-like receptor 7 (TLR7)-activated pDCs (68). pDCs show quite different functions in TME, depending on its immature or mature state. Mature pDCs acquire dendritic morphology, upregulate MHC and T cell costimulatory molecules, and produce high amounts of IFN-α (69), which can affect tumor cell proliferation and tumor metastasis (70), while immature pDCs cannot demonstrate the similar functions. With higher level of BDCA2 expressed in peritumoral and tumoral tissues, we speculated that the majority of pDCs in these tissues might belong to immature state. Similar to mechanisms previously shown in tumor cells (71–73), recent study conducted by Basit et al. showed that toll like receptor (TLR)-stimulation changed expression of genes regulating oxidative phosphorylation (OXPHOS) and glutamine metabolism in pDCs, while inhibition of glutaminolysis and OXPHOS prevented pDCs activation (74). Previous studies have also shown that immature pDCs recruited to the tumor site mediated by the interaction of CXC chemokine receptor 4 (CXCR4), resulting in promotion of pDCs-mediated generation of CD4^+^CD25^+^Foxp3^+^Treg cells, which leads to anergy and immune suppression, favoring the immune escape of tumor cells (75, 76). Hence, our current study has found a positive correlation between BDCA2^+^pDCs and Foxp3^+^Tregs in stomach tissues, which is consistent with the findings performed by Geva-Zatorsky and colleagues (8). In addition, several studies has found that the prognosis of different types of tumors is inversely related to the number of tumor-infiltrating pDCs (25, 26).

Tregs, one kind of tumor-infiltrating lymphocyte, played a crucial role in maintaining immunological self-tolerance when the immune system eradicated tumor cells. Previous studies have found that high density of tumor-infiltrating Tregs was accumulated in various human carcinomas (77–81). The function of these Treg cells has been described to suppress proliferation, induction/activation of CD8^+^ and CD4^+^ T cells in a COX-2-dependent manner, which is involved in establishing immunological tolerance in cancer immunosuppression (82). Treg-mediated immunosuppression has been considered as one of the crucial immune evasion mechanisms in tumor whereby they are able to overcome the anti-tumor activity of CD8^+^ cytotoxic T cell, DCs and natural killer cell (83). Different Treg subsets can produce different inhibitory cytokines and induce different lymphocytes, endowing Tregs with different functions. Increasing evidence shows that the transcription factor Foxp3 expression plays a key role in Treg-mediated dominant suppression and that Foxp3 expression significantly contributes to invasive breast cancer by tipping the balance toward a more immunosuppressive microenvironment (84–86). Yuan et al. has found that elevated Foxp3 expression in tumor-infiltrating Treg cells was associated with the TNM stage in GC patients (82). Previous study has also found that intratumoral Foxp3^+^Tregs/CD8^+^T ratio was an independent predictor for the prognosis of GC, with higher Foxp3^+^Tregs/CD8^+^T ratio representing worse overall survival (87). Our present study found that higher levels of Foxp3^+^Tregs were observed in peritumoral and tumoral tissues than that in normal tissue. Different from the alterations of BDCA2^+^pDCs, there was no significant differences of Foxp3^+^Tregs between peritumoral and tumoral tissues, which indicated that peritumoral and tumoral tissues showed similar TME that could not effectively prevent the invasion of tumor cells.

Both higher BDCA2^+^pDCs and Foxp3^+^Tregs in peritumoral and tumoral tissues demonstrated the disturbance of gastric mucosal immunity homeostasis. Although commensal bacteria were crucial in maintaining immune homeostasis of the intestine, the roles of gastric microbiota on gastric mucosal immunity regulation were still unclear. Our present study found that the gastric microbiota was not uniform among the different microhabitats in the gastric TME, with decreased diversity in peritumoral tissue and decreased species richness in peritumoral and tumoral tissues, while the composition of the gastric microbiota changed significantly in tumoral tissues. Geva-Zatorsky et al. has demonstrated that microbial diversity in the gut ensures robustness of the microbiota's ability to generate a consistent immunomodulatory impact, serving as a highly important epigenetic system (8). Previous studies have found that the increased abundance of HP in the tumoral sites is classically believed to contribute to GC tumorigenesis, and mass eradication of HP significantly decreases the risk of developing cancer in infected individuals without pre-malignant lesions (88–90), reinforcing the theory that HP influences early stages in gastric carcinogenesis. However, our present study only found higher relative abundance of HP in peritumoral tissue than that in normal and tumoral tissues, but no correlations between HP and gastric immunosuppression. This might be associated with its “hit and run” mechanism for GC development, which HP is no longer present in the intratumoral microhabitat at the time GC is identified (91). The changed hypochlorhydric mucosal environment in tumoral tissue was not suitable for HP colonization. Although HP was not correlated with immunosuppressive cells, its colonization could also affect the overall structure and composition of the gastric microbiota, which might affect the antitumor immunity and participate in GC development indirectly. Our study found that many non-dominant gastric bacteria were significantly correlated with the two major immunosuppressive cells. Most of these bacteria belonged to the phyla Proteobacteria and Firmicutes, which modulated gastric mucosal immunity alone or in together with its bacterial metabolites. Fujiwara et al. described a reduction in pDCs in mice with a restricted microbiota distinct from that typical of specific pathogen-free mice (92), whereas Dasgupta et al. revealed induction of pDCs in mesenteric lymph nodes by *B. fragilis* during ongoing colitis (93). Recently, Geva-Zatorsky et al. has found that the frequency of pDCs in colon and small intestine is influenced by gut microbiota but with great variable in germ-free mice monocolonized with one commensal bacterial strain. They found that the transcripts (from the colons of monocolonized mice) most correlated with pDCs frequency were enriched in lipid or protein digestion and metabolic pathways, an observation suggesting a connection between pDCs and the metabolic and nutrient uptake functions of the gut (8). Previous study found that *Stenotrophomonas* was associated with a wide spectrum of diseases and substantial morbidity/mortality in immunosuppressed patients with cancer (94). *Stenotrophomonas* showed immunostimulatory properties, inducing TNF-α expression and contributing significantly to inflammation, which might participate in TME modulation in the stomach. Our present study firstly demonstrated that *Stenotrophomonas* was positively correlated with BDCA2^+^pDCs. *Comamonas* was a cellulolytic microbe, which could impact the host metabolism in cancer patients and are associated with inflammation (95). Recently, Garcia-Gonzalez et al. has demonstrated that *Comamonas* could decrease 5-fluoro-2'-deoxyuridine efficacy, but increase the efficacy of the topoisomerase I inhibitor camptothecin (96). In our present study, we found that *Comamonas* was negatively correlated with BDCA2^+^pDCs, which might play a role in antitumor immunity. Our previous study observed that *Selenomonas* was enriched in patients with colorectal cancer and almost absent from healthy individuals (35). In GC patients, we also found higher relative abundance of *Selenomonas* in peritumoral and tumoral microhabitats, which exerted local effects on the immune system and was correlated with Foxp3^+^Tregs positively. In addition, a new microorganism often isolated from soil, *Gaiella*, also showed immunostimulatory properties and was negatively correlated with gastric Foxp3^+^Tregs through an unknown mechanism. Our present study found that the functional content of the gastric microbiota changed significantly in tumoral tissue, especially in metabolism-related pathways. Overall, the impaired gastric mucosal immunity might be the results of the interplay between the gastric microbiota and the stomach, which lead to tumor escape from immune surveillance.

In summary, we firstly demonstrated the relationship between gastric mucosal microbiota in different microhabitats and its corresponding gastric mucosal immunity in GC patients. The recruitment of two major immunosuppressive cells, BDCA2^+^pDCs and Foxp3^+^Tregs, represented the disturbance of gastric mucosal immunity homeostasis in the gastric TME that contributed to the GC development. The structure, composition and function of the gastric mucosal microbiota changed significantly among different microhabitats. Interestingly, we found that the gastric microbiota especially those non-abundant genera was correlated with BDCA2^+^pDCs and Foxp3^+^Tregs significantly. Our findings implied that the gastric microbiota might participate in modulating tumor-immune microenvironment actively, which indicated that gastric microbiota might be a key orchestrator of cancer therapy. Our present study will provide new insights on personalized GC diagnosis and treatment by modulating TME by gastric microbiota.

## Ethics Statement

The present research was approved by the Ethics Committee of the First Affiliated Hospital, School of Medicine, Zhejiang University (Zhejiang, China). Informed written consent was obtained from each of the patients before enrollment.

## Author Contributions

ZL and XsL conceived and designed the experiments. ZL, XsL, LS, XL, YC, YM, FJ, and CY performed the experiments. XsL, LS, and ZL analyzed the data. ZL, LS, and XsL wrote the paper and edited the manuscript. The final manuscript was read and approved by all authors.

### Conflict of Interest Statement

The authors declare that the research was conducted in the absence of any commercial or financial relationships that could be construed as a potential conflict of interest.
